# Cnidarian Interaction with Microbial Communities: From Aid to Animal’s Health to Rejection Responses

**DOI:** 10.3390/md16090296

**Published:** 2018-08-23

**Authors:** Loredana Stabili, Maria Giovanna Parisi, Daniela Parrinello, Matteo Cammarata

**Affiliations:** 1 Istituto per l’Ambiente Marino Costiero, U.O.S. di Taranto, CNR, Via Roma 3, 74123 Taranto, Italy; 2 Dipartimento di Scienze e Tecnologie Biologiche ed Ambientali, Università del Salento, via Prov.le Lecce Monteroni, 73100 Lecce, Italy; 3 Laboratory of Marine Immunobiology, Dipartimento delle Scienze della Terra e del Mare, Università di Palermo, Viale delle Scienze Ed. 16, 90128 Palermo, Italy; mariagiovanna.parisi@unipa.it (M.G.P.); daniela.parrinello@unipa.it (D.P.)

**Keywords:** cnidarian, anthozoa, microbial communities, cnidarian holobiont, zooxanthellae, bleaching, antibacterial activity

## Abstract

The phylum Cnidaria is an ancient branch in the tree of metazoans. Several species exert a remarkable longevity, suggesting the existence of a developed and consistent defense mechanism of the innate immunity capable to overcome the potential repeated exposure to microbial pathogenic agents. Increasing evidence indicates that the innate immune system in Cnidarians is not only involved in the disruption of harmful microorganisms, but also is crucial in structuring tissue-associated microbial communities that are essential components of the Cnidarian holobiont and useful to the animal’s health for several functions, including metabolism, immune defense, development, and behavior. Sometimes, the shifts in the normal microbiota may be used as “early” bio-indicators of both environmental changes and/or animal disease. Here the Cnidarians relationships with microbial communities and the potential biotechnological applications are summarized and discussed.

## 1. Introduction to Cnidarian

**Figure 1:**
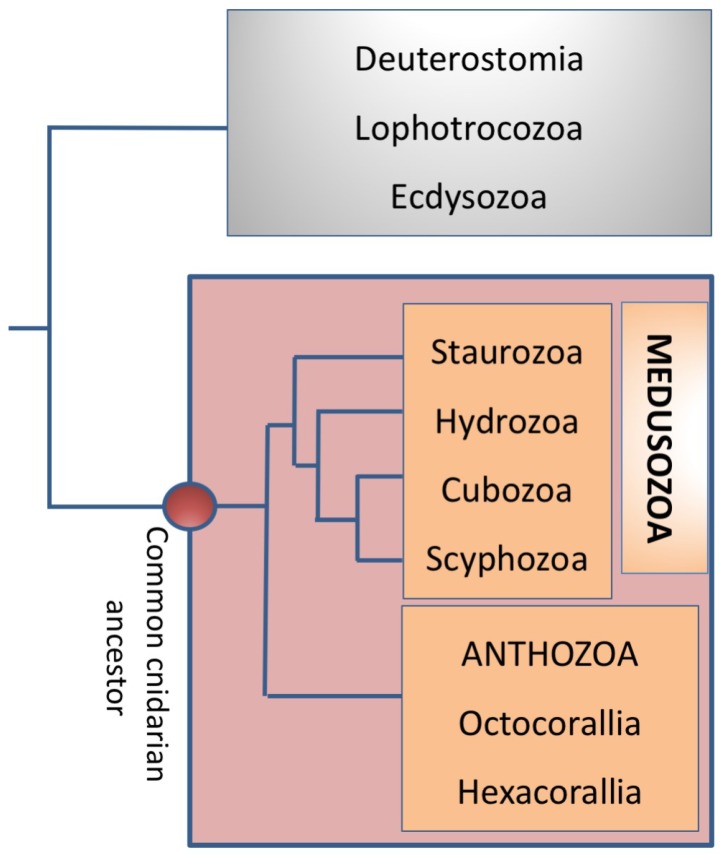
The phylogenetic relationships of Medusozoa (Staurozoa, Hydrozoa, Cubozoa, and Scyphozoa) and Anthozoa as reported by Boero et al. [1]. Molecular data sustain the separation in two class of the Anthozoa, which are common distinguished by tentacles morphology. Octocorallia is a group of hard coral species living at a depth of more than 100 m. This is a very slow growing species, formed by polyps with eight tentacles which capture floating materials of up to several hundred microns and included soft corals. Hexacorallia is a group of several hundred reef-building coral species including stony coral and sea anemones. The polyps of this coral have tentacles in groups of six, instead of eight.

Cnidarian are a group made up of more than 9,000 living species, exclusively aquatic, getting their name from the presence of cnidocysts connected to supporting cells and neurons. These in turn form a unique chemosensor and mechanoreceptor neuronal cell complex that releases highly-ordered secretion products upon stimulation. The phylum Cnidaria includes the corals, hydras, jellyfish, Portuguese men-of-war, sea anemones, sea pens, sea whips, and sea fans. Cnidaria are taxonomically subdivided into: Anthozoa (Hexacorallia and Octocorallia) with the absence of a medusa stage, and the Medusozoa, that usually exhibit a medusa stage in their life cycle and includes the classes Cubozoa, Hydrozoa, Scyphozoa, and Staurozoa [1] (Figure 1).

The Cnidaria are one of the earliest branches in the animal tree, with tissue layers, muscles, and sense organs. They are diploblastic, have a radial symmetry, do not possess a real brain having only two cell layers; the epithelial cells are involved in all the innate immune responses. The endodermal epithelium functions as a chemical barrier using antimicrobial peptides, while the ectodermal epithelium represents a physicochemical barrier. Furthermore, Cnidaria are present in the fossil record since the Precambrian, when the other animals similar to the present ones were absent [1,2].

Several important issues related to immunity can be inferred from the diversity in cnidarian life histories and habitats. In particular, in some cases their life cycles are very long and they may be subjected to repeated exposure to pathogenic agents [2]. Consequently, in the absence of specific immune cells, cnidarians must have effective mechanisms to defend against microbial pathogens. Furthermore, colonial forms, in order to save tissue integrity, rely on their capacity of self/nonself discrimination to rapidly recognize approaching allogeneic cells as foreign and to remove them [3]. Finally, successful growth for cnidarians is related to their capacity to differentiate between beneficial symbionts and pathogenic intruders [4,5], since they are colonized by complex bacterial communities and in several cases constitute home to algal symbionts. On account of these considerations it is of interest to understand how animal’s longevity have modified the defense to innate components of immunity, leading us to consider Cnidaria as good candidates at the crossroad of metazoan evolution. Several molecular “omics” studies on the hydrozoans [6,7] sea anemones [8,9] and corals [8,10,11] demonstrated that some genes, associated with the immune responses, resulted conserved from cnidarians to vertebrates.

## 2. Cnidarians Associated Microbial Communities

Marine microorganisms are present at high density representing a major component in terms of the biomass on Earth. By the advent of the powerful tools of the molecular biology, remote sensing, and deep sea exploration, amazing discoveries on the abundance and diversity of marine microbial life and its function in global ecology have been made. In particular, researches on the relationships of microbial components with other organisms have furnished new information on the phenomena of food networks, symbiosis and pathogenicity [12,13].

**Figure 2:**
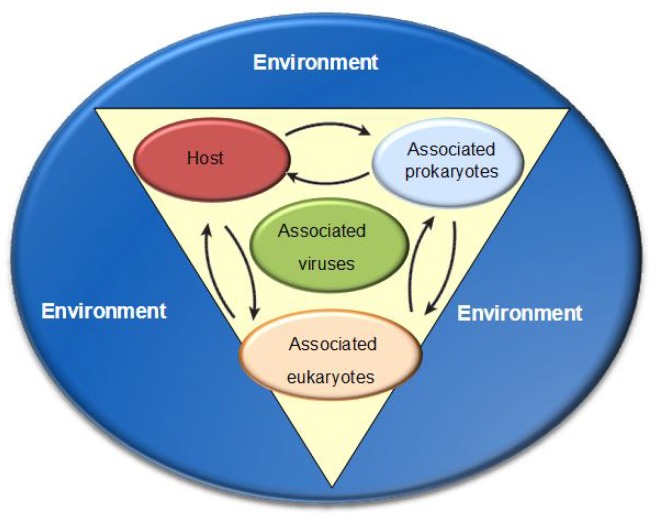
Multicellular organisms as “metaorganism” including the macroscopic host and the synergistic associated bacteria, archaea, fungi, and numerous other microbial and eukaryotic species. Modified from Bosch T.C. and McFall-Ngai M.J. 2011.

Recently, there has been increasing interest in microbes as a relevant portion of the animal phenotype, responsible for the fitness as well as the ecological features of their hosts [14,15]. Several studies accomplished by genetic and genomic approaches have provided evidence for several animal–bacteria interactions in invertebrates and vertebrates revealing that bacteria play a crucial role in facilitating animals’ origin and evolution [16,17]. Moreover, these findings clarified that animals and bacteria mutually influence their genomes [18] and that the homeostasis between animals and their symbionts is maintained by complex mechanisms [19,20]. Considering that microbial communities colonize all epithelia in animals, each animal with its associated microbes can be can be treated as a metaorganism (Figure 2) composed of the macroscopic host and the mutual symbiotic association with bacteria, archaea, fungi, and other microbial and eukaryotic species [21]. In such a community, membership is often influenced by interactions among species and properties [22].

In the case of Cnidarians, associated protists, bacteria, archea, and viruses are essential components of the Cnidarian holobiont, capable of influencing, for example, the health of corals and coral reef ecosystems [21,23,24]. The associated bacteria perform several potential roles, such as nitrogen fixation, antibiotics synthesis [25,26], organic compounds decomposition [27], and space utilization; avoiding pathogen colonization [28]. Complexity and diversity are peculiar characteristics of coral-associated bacteria which reveal host species-specificity [29,30] and differ when compared to the bacterial communities recorded in the surrounding seawater [29,31,32]. Coral-associated microbial communities are influenced in their composition by several ecological parameters. When changes in environmental parameters are recorded, e.g., increases of seawater temperature, microbial species change in their density, making the coral holobiont capable of adapting to the new condition. Most studies have been carried out conclude that bacteria are directly involved in coral diseases [23,24,30,33,34]. Microbial communities associated with corals constitute a key factor useful to understand the coral reef health. Changes in bacterial composition over time may influence coral health and consequently their sensitivity to disease. Some researchers [35] have shown that when a small portion of the colony exerts signs of disease, the bacterial community associated with the colony is affected and modified. As a consequence, these data indicate that the evaluation of the shifts in the normal microbiota may be employed as “early” bio-indicators of both environmental changes and coral disease. Stress due to anthropogenic activities as well as environmental impacts may result in changes in the coral-associated microbial communities reflected as negative effects on the entire coral [28]. Climate change has been indicated as one of the foremost threats to Indo-Pacific reefs strictly related to coral bleaching. In some instances, at high temperatures, certain bacterial species increase their virulence and have been considered involved, for example, in bleaching [36]. It is therefore possible that the disappearance of key bacterial associates (by biotic or abiotic disturbances) amongst these communities provide an entry niche for opportunistic species that can further interfere with the microbial community structure and health status of the coral holobiont [35].

In particular, studies on the coral *Oculina patagonica* in the Mediterranean Sea have shown that the causative agent of the bleaching disease (resulting in the expulsion of the endosymbiotic zooxanthellae) is *Vibrio shiloi* [37]. In association with the onset of bleaching this bacterium exerts some virulence factors when high temperature values are recorded [38,39]. Moreover, *Vibrio coralliilyticus* was responsible for the bleaching of the coral *Pocillopora damicornis* on the coral reefs in the Indian Ocean and Red Sea [40]. In some coral diseases, such as black band, white pox, and white plague, bacteria are involved [33,36,41] and more than twenty coral diseases have been described (Table 1: from Rosenberg et al. [42]).

These diseases and their etiological mechanisms have been widely investigated over several years [43]. However, Reshef et al. [44] indicated that *O. patagonica* has become resistant against the infection supported by *V. shiloi*; thus, this bacterial species can no longer be isolated on the corals, and this *Vibrio* species, previously infecting corals is unable to produce disease on the existing corals. In order to explicate these results Reshef et al. [44] proposed the “Coral Probiotic Hypothesis”. The term ‘probiotic’ means ‘for life’ and is referred to live microorganisms capable of determining a benefit in terms of health on their host [45,46]. Thus, these invertebrates seem not only to tolerate, but also to need the colonization by beneficial microorganisms for several functions including metabolism, immune defense, development, and behavior [47,48,49].

## 3. Tissue-Associated Microbial Communities

As suggested by the increasing evidence the innate immune system in Cnidarians is not only involved in the disruption of harmful microorganisms, but also plays a crucial role in maintaining those tissue-associated microbial communities, useful to the host’s health [50,51]. This is also the case with Hydra. Bacteria are indeed an important component of the Hydra holobiont in which 36 bacterial phylotypes were identified belonging to three different bacterial divisions and are dominated by the phyla Proteobacteria and Bacteroidetes [52]. The health of the whole animal can be compromised by disturbances or shifts in any of these partners [53]. In laboratory studies Hydra have been cultivated under standard conditions (constant temperature and identical food), and surprisingly, it was observed that in different Hydra species, maintained in the laboratory for more than 20 years, a complex microbial community colonized the epithelium which greatly differed in individuals from different species, but was cultured under identical conditions. On account of these evidences it was concluded that the microbiota in Hydra is specific for each species [12].

When closely related Hydra species were examined, the associated microbial community that resulted was similar. This, for instance, was the case of *H. vulgaris* and *H. magnipapillata*. Moreover, the early branching linage of Hydra species *H. oligactis*, examined so far [54], results associated with the most distinct microbial community in comparison with the other Hydra species. These observations lead to conclude that on and within the Hydra epithelium distinct selective pressures are imposed [50]: the colonization of a certain epithelium by bacteria is related to several ecological factors, such as host immune responses, the availability of nutrients, and the space competition between bacterial strains. Thus, it can be suggested that the colonizing microbial composition is shaped by both host factors such as components of Hydra’s innate immune system and frequency-dependent bacteria–bacteria interactions [51].

In contrast to microbiomes of tropical corals, characterized by high diversity, Mediterranean octocorals harbor structured bacterial assemblages in which only a few species prevail for >90% [55,56]. This makes them ideal model organisms to investigate cnidarian microbe interactions. In the Mediterranean Sea, gorgonians contribute significantly to the structural complexity, biomass, and biodiversity of these ecosystems constituting the most significant habitat-forming species of benthic communities [19]. The success of gorgonians is in part due to the specific symbioses with bacteria which are relatively stable across spatial scales. Bacteria belonging to the genus Endozoicomonas are the most prevalent [57,58] in certain species from the Gorgoniidae family. Endozoicomonas have been recorded in several marine invertebrates and have been recognized as crucial to the health of corals [59], with a loss of these microorganisms producing a conspicuous negative impact on the holobiont functioning. Spirochaetes represent one of the (co-)dominant microbial associates [60], presumably involved in nitrogen and carbon fixation in tropical octocorals and deep-sea gorgonians, particularly the precious red coral *Corallium rubrum* [61]. The spatial stability of these bacteria–host associations, which may exist in in the same habitat and location, lead to hypothesize strong selection mechanisms used by the holobiont of Mediterranean gorgonians.

Therefore, exploring the structure and functioning of the microbiome is a major challenge of current research in Cnidarians, also taking into account that their tissue and mucus support a diverse microbial community [24,34,62].

## 4. Mucus-Associated Microbial Communities

Mucus adhesion and colonization by bacteria represents one of the best characterized symbiosis in the marine environment. Specifically, mucus released by certain marine invertebrates furnishes a habitat for several bacteria [63,64]. During mucus production, marine organisms consume a significant portion of energy. For instance, in corals mucus release consumes up to 50% of the assimilated energy [65]. Some marine invertebrates are coated by a layer of mucus to prevent bacteria and debris from accumulating on the body surface [66]. This matrix is involved in a number of defense mechanisms [67,68,69,70,71,72], to cope with the rich mixture of microorganisms in the surrounding water. However, for many species, including corals, as reported by Coffroth [73], the mucus also represents a home site [63,64] and may function as a potential food source [74]. Mucus contains primarily polysaccharides and proteins with C:N ratios of 1:5, on account of this composition it was suggested that this matrix, released by some marine invertebrates, is readily degradable by microbes, thus supporting microbial growth. As regards Cnidarian, mucus contains many microorganisms [24,75,76], and in particular, in the coral mucus, the mean concentration of colony-forming bacteria is about 0.2% of the total counts determined microscopically by using SYBR Gold Staining and ranges between 10^5^ and 10^6^ mL^−1^ [77,78]. Furthermore, in several other corals, including the Caribbean coral *Monastrea franks* and *Oculina patagonica* [28,79], the mucus layer was shown to contain a high bacterial density (3 × 10^8^ mL^−1^). Species belonging to the three primary domains Archaea, Eubacteria, and Eukarya have been recorded in coral mucus [80]. In *H. vulgaris* mucus *V. splendidus* was the most-abundant species attaining 68% and 50% in the winter and summer, respectively [78]. Moreover, vibrios prevailed in the culturable bacterial isolates from the mucus of *Acropora palmata*. Most of the studies have conducted on the microbiota associated with corals and other marine invertebrates’ mucus [76,81], by contrast, zoanthids have received little attention in that respect, although only few studies by Chimetto et al. [75,82,83] investigated the diversity of bacteria on zoanthids and found 16S rDNA sequences belonging to the *Vibrio* genus. As already proposed by Calow [74], differences in biochemical composition may render the mucus more or less susceptible to microbial attack. The mucus rich in proteins released by some invertebrates is rapidly used by microbes possessing exoenzymes potentially capable to degrade mucoid polymers [73,84]. On account of this activity, microbial communities harbored in such mucus may use mucus-derived, dissolved, and particulate matter transforming them into living biomass [84]. As a consequence, the mucus can represent the foundation from which microbial organisms are then preyed upon by other organisms [85,86,87,88]. In contrast to this, some mucus bacteria are involved in the defense of their hosts by the production of antibacterial compounds. In turn, mucus bacteria capable of producing antibacterial molecules have an advantage over other microorganisms, assisting in competition over space and nutrition. Several bacteria with antimicrobial activity against presumed coral pathogens have been isolated from corals [26,89,90], and the antimicrobial activity of coral mucus appears decreased in corals displaying signs of coral bleaching or disease [26]. These observations suggest intriguing relationships between different coral-associated bacteria and between bacterial associates and the coral host. It is currently unknown whether bacterial communities are selected by extrinsically mediated factors or whether the holobiont itself selects for beneficial associates [90]. Analogous studies of sponges hint at the latter hypothesis as possible, where it is suggested that the species *Mycale adhaerens* may selectively sequester bacterial epibionts with antimicrobial activities [91]. One major group of coral-associated bacteria exerting antibacterial activity is *Pseudoalteromonas* sp. Several *Pseudoalteromonas* produce antibacterial compounds, toxins, bacteriolytic substances, and enzymes, all of which may aid the bacterial cells in their competition for space, nutrients, and in the security from predation [92]. It is also plausible that bacteria such as *Pseudoalteromonas* sp. can affect the microbial community by releasing active compounds into the coral mucus. This is in accordance with the “Coral Probiotic Hypothesis” [44], whereby active *Pseudoalteromonas* sp. can be considered as “probiotic” to corals, taking part in coral holobiont defense against bacteria.

Extraordinary recent progress in sequencing technologies and the ability to culture simple but genetically accessible model organisms for some time under germ-free conditions are revealing details of host–microbe interactions that highlight the value of an evolutionary perspective thus undermining prior concepts. However, in spite of these insights, the factors involved in microbial colonization of mucosal surfaces are still unknown. Moreover, the accumulated data are not still coherently integrated in order to obtain a truly mechanistic understanding of host–microbe interactions on host mucosal surfaces.

## 5. Innate Immune System as a Regulator in Maintaining Homeostasis between Animals and Their Resident Microbiota?

In a recent review, Bosch [50] has reviewed the pre-existing idea that immune systems evolve exclusively to control invading pathogens furnishing evidence that host-specific microbiota is established by the crucial role played by major factors of the immunological system. The involvement of components of the innate immunity systems, such as antimicrobial peptides, in shaping the microbiota is now undeniable. His thesis, based mainly on Hydra examples, is that the need to control of the resident beneficial microbes induced the evolution of the immune systems. He suggested that it is reasonable to assume that the inferences drawn apply to both invertebrates and vertebrates. Stem cell proliferation, microbiota composition, and innate immunity seem to have a mutual direct link. Particularly, he highlighted that recent discoveries in Hydra show that homeostasis between animals and the resident microbiota is assured by the action of innate immune system factors and transcriptional regulators of stem cells. He stated that, in early-branching metazoans, the evolution of the innate immune system and its host-specific components is due to the need to control the resident beneficial microbes, rather than the action of invasive pathogens. In this framework, disease onset is considered as the result of a complex network of interactions among different associated partners capable of affecting the fitness of the entire metaorganism [93].

This is also the case of microbial hypothesis of coral bleaching. According to this hypothesis, several physical and biological factors, including variation in sea surface temperatures [94,95], UV irradiation [96], low salinity and pollution [97], and bacterial infection [43] are responsible for bleaching, which is a symptom of stress. These different kinds of stress act on both the coral microorganisms and the coral host, determining a change in the microbial community that in some cases is reflected directly or indirectly on bleaching. This also [98] emerges from research conducted over the last decade, which has supported that the coral host, its endosymbiotic zooxanthellae, and a large number and variety of accompanying microorganisms form a complex and dynamic symbiosis represented by the coral holobiont. In a healthy coral, the growth, reproduction, and disease resistance of the holobiont is due to the metabolic activities of each organism interacting with the other ones. Thus, the coral host, by capturing and feeding on prey, through their digestion, provides nutrients for its associated microorganisms. The associated microorganisms may be also used directly by the coral [99] with the production of carbon dioxide and water as byproducts of cellular respiration. The zooxanthellae, in turn, employing the carbon dioxide and water, accomplishes photosynthesis. In particular, *Symbiodinium* reside in host tissues at millions of cells per square centimeter and provide the energy required by reef building corals to grow, calcify, and reproduce. The zooxanthellae cells produce, as products of photosynthesis, sugars, lipids (fats), and oxygen; major components needed for animal and bacterial respiration. Thus, the driving force behind the growth and productivity of coral reefs is represented by the tight recycling of products between the polyp cells, bacteria, and the zooxanthellae. Under stress, the components of the symbiosis separate and the associated endosymbionts may be digested [100]. In this framework, the roles of bacteria in contributing to the holobiont health is a matter of current interest and debate. It has been shown that coral bacteria can fix nitrogen, degrade complex polysaccharides, and produce antibiotics useful in helping to prevent infection by pathogens. Rosenberg et al. [98] suggested that coral bleaching happens when the equilibrium between the different components of the coral holobiont is destroyed and results in a decrease in the endosymbiotic zooxanthellae. Bleaching is now considered a host innate internal defense response to compromised symbionts and, in particular, Cnidarian bleaching is due to a breakdown in the symbiotic relationship between host cnidarians and photosynthetic dinoflagellates belonging to the genus *Symbiodinium*. The symbiosis between anthozoan polyps and zooxanthellae are considered nonharmful infections, where the unicellular organisms are able to control the host defense response until the environmental conditions are optimal for survival of autotrophic and heterotrophic organisms. The oxygen reactive species (ROS) and the reactive nitrogen species nitric oxide (NO) are involved in host–pathogen interactions and bleaching events. The stress triggered by alterations of physical factors, pathogenic infections, or injuries indeed involves the increase of ROS and NO by the symbionts. These molecules activate the cascade mechanisms in internal defense systems and eliminate the zooxanthellae. The loss of the symbionts unable to perform the photosynthesis process occurs through traditional mechanisms of the innate immune system including exocytosis, host cell detachment, and apoptosis [101]. Although bleaching is induced by a variety of environmental stressors like global climate change and high solar radiation, the temperature increase in the superficial seawater and anthropogenic stress also caused an enhancement in diseases of species of the genus Anthozoa responsible for the bleaching or tissue death [102]. However, most works have concentrated on the innate immune repertoire of anthozoans, the immune effector mechanisms mediated corals adaptation to such events remain almost unknown.

## 6. Antimicrobial Peptides, Multifunctionality, and Biotechnological Implications

Humoral response is realized by the synthesis and release of an array of chemical compounds, including melanin, reactive oxygen species (ROS), antimicrobial peptides (AMP), and secondary metabolites whose most important purpose is to destroy microbes by: (1) opsonizing and agglutinating invaders; (2) permeabilizing the invader’s cell membrane, causing lysis; or (3) disruption of their metabolism [103]. Antimicrobial agents do not possess functional specificity since exert a broad spectrum of activity against Gram-positive and Gram-negative bacteria, fungi, viruses, and protists [103,104]. However, all antibacterial agents are not equally effective toward bacteria [105]. The size of the antimicrobials may impact structural microbial components differently. For instance, small peptides (<23 amino acids long) mainly destroy the cell membrane integrity of the invaders, while larger peptides pose lytic properties, or may be proteins with specific domains that sequester essential nutrients from microbes [106]. Several studies have been attained to evidence the presence of antimicrobial compounds in cnidarians. The antimicrobial activity of the eight species of gorgonian corals *Plexaura homomalla*, *Pseudoplexaura flagellosa*, *Plexaurella fusifera*, *Eunicea clavigera*, *Eunicea tourneforti*, *Eunicea laciniata*, *Eunicea calyculata* (Plexauridae), and *Pseudopterogorgia americana* were assayed against five species of bacteria including marine bacteria as well as human pathogenic species (*Vibrio harveyi*, *Pseudomonas aeruginosa*, *Serratia marcescens*, *Staphylococcus aureus*, *Bacillus megaterium*, and *Escherichia coli*). Antimicrobial activity was evaluated on polar and nonpolar extracts and was most apparent in the nonpolar fractions. In general, marine bacteria were not as sensitive to the extracts as the nonmarine species [107]. Subsequently, from the West Indian gorgonian coral *Pseudopterogorgia elisabethae*, by using NMR spectroscopy, the structure of two compounds capable to inhibit the growth of *Mycobacterium tuberculosis* H37Rv t. was determined [108]. The activity was ascribed to two diterpenoid alkaloids, namely pseudopteroxazole, producing a 97% growth inhibition, and seco-pseudopteroxazole, responsible for a 66% inhibition at 12.5 μg/mL. From the same octocoral *P. elisabethae* of San Andrés and Providencia Islands (Southwest Caribbean Sea) [109] the cytotoxic and antimicrobial activity of pseudopterosins and secopseudopterosins, active against *Staphylococcus aureus* and *Enterococcus faecalis* but inactive against *Pseudomonas aeruginosa* and *Candida albicans*, was investigated. Shapo et al. [110] reported that crude extracts from the gorgonian coral *Leptogorgia virgulata*, likely containing homarine, showed inhibitory activity against *Escherichia coli* and *Vibrio harveyi* as well as other bacteria. Uncharacterized antimicrobial agents have been also documented in over a dozen members of the Plexauridae, Gorgonidae, and Ellisellidae families [107,111,112]. Chen et al. [113] reported that fifteen guaiazulene-based terpenoids (anthogorgienes A–O) and eight analogues, isolated from the lipophilic extract of *Anthogorgia* sp., were effective against *S. aureus* and *Streptococcus pneumoniae* and three fungi (*Aspergillus fumigatus*, *Aspergillus flavus*, and *Fusarium oxysporum*). In the sea-whip *Dichotella gemmacea* Li et al. [113] isolated six briarane diterpenoids and two analogs showing a weak antimicrobial action against the growth of *E. coli*. Scleractinian corals also possess secondary compounds with antimicrobial properties, even though they have been less well investigated than those derived from the gorgonians [114]. Gochfeld and Aeby [114] reported that crude extracts from three Hawaiian corals i.e., *Montipora capitata*, *Porites lobata*, and *Pocillopora meandrina*, exerted antibacterial activity against coral pathogens such as *Serratia marcescens*, *Vibrio coralliityticus*, and *V. shilo*. However, the antibacterial activity of extracts varied among species and as a function of the state of health of the host. The antimicrobial properties of extracts from Red Sea soft corals (alcyonaceans) *Litophyton arboreum*, *Rythisma fulvum*, *Heteroxenia fuscescens*, *Sarcophyton glaucum*, *Dendronephthya hemprichi*, and *Xenia macrospiculata*, were compared with those of stony (scleractinian) corals, *Acropora variabilis*, *Fungia scutaria*, *Fungia granulosa*, *Turbinaria* sp., *Stylophora pistillata*, and *Favia favus* and the majority of soft corals (83%) resulted to affect remarkably the growth of the marine bacteria *Arthrobacter* sp. and scarcely the growth of *Vibrio* sp., while stony corals showed very little or no activity [115].

In recent studies, thermostable proteases and antimicrobial peptides have been characterized from the body and tentacles of the sea anemones *Actinia equina* and *Anemonia sulcata* with application for biocleaning and as antifungals [115]. In particular, bioactive molecules (BMs) isolated from the sea anemone *Actinia equina*, were proven to hydrolyse aged/altered protein layers or coatings as well as to control bacteria/fungi growth [116]. On account of these features these molecules represent an innovative tool in conservative restoration procedures. Particularly, the BMs molecules with proteolytic activity were tested in order to remove protein layers or to control microbial colonizations. The removal of undesired layers under “room temperature” (19–25 °C) conditions, without heating the enzyme solution or the artwork surface on which it was applied was tested. Agreeable results were obtained after application of gelled enzymatic solution, in removing coherent protein layer both from the surface of polychrome wood or wax sculpture. In both cases the complete removal of the protein layer, without producing whitening phenomena was observed. The best advantage of these molecules is their temperature of action (<30 °C) which is different from that of the commercial proteases active at higher temperature (37 °C). Moreover, the antimicrobial activity of BMs was assayed to inhibit the growth of some bacteria such as *Enterobacter* spp. and *Micrococcus luteus*, and fungi as *Aspergillus niger* and *Penicillium chrysogenum*. Thus, the employment of these molecules for biocleaning represents an innovative procedure that minimizes the exposure to harmful solvents and chemicals compounds for both the workers and the environment [116]. Furthermore, these molecules are totally safe for works of art, restores, and the environment, requiring a short time of application. Consequently, we hypothesize that these bioactive molecules represent a valid alternative to the traditional procedures in sustainable restoration projects [116].

Moreover, recently it was established that anthozoans could also benefit of the multifunctionality of some of their bioactive molecules [117]. *Actinia viridis* and *Actinia equina* possess a toxin with bifunctional characteristics: Neurotoxin ATX II, isolated from *A. viridis*, is a sodium channel type 1 toxin constituted of 47aa, characterized by the presence of three disulfide bridges capable of binding to the sodium voltage ionic channel, delaying the inactivation phase during the transmission of action potential and exerting antimicrobial activity towards *Micrococcus lysodeikticus*. ATX II can be considered as a neurotoxin with an additional antimicrobial peptide property. Thus, anemones could adopt the multifunctionality of toxins as an evolutionary strategy in order to amplify their predation capacity. Moreover, the antimicrobial molecules would assure the polyps to survive avoiding bacterial infections [117]. *Actinia equina* lives in the temperate coastal area and this intertidal species is a suitable and exemplary model for the study of bioactive molecules and their evolution. Hemolytic molecules such as equinotoxin [118,119] and proteins for potassium and sodium voltage dependent channels [120] have been characterized. The mucus of this sea anemone contains a complex mixture of proteins and polysaccharides with differential biological activity implicated in the immune defense. This matrix plays a crucial role in a series of biological processes including structural support, locomotion, food particle trapping, and defense against multiple environmental stresses, predators, parasites, and pathogens. In this mucosal matrix hemolytic activity versus rabbit erythrocytes, cytotoxic activity against human erythromyeloblastoid leukemia T cell line (K562) and lysozyme-like activity was observed [71]. Lysozyme is involved in internal innate defense and acts as an antimicrobial enzyme system and in particular as a glycoside hydrolase, catalyzing the hydrolysis of 1,4-beta-linkages between *N*-acetylmuramic acid and *N*-acetyl-d-glucosamine in peptoglycans component of bacterial cell wall. As a consequence, the integrity of bacterial pathogens through the lysis of their cell wall results compromised. The presence in *A. equina* mucus of an antibacterial activity in association with a hemolytic and cytotoxic activity indicates its participation in the defense system against pathogenic invaders suggesting that the humoral effectors of the internal defense system can be released in mucus layer. The activity against *Micrococcus lysodeikticus* as well as the satisfactory results obtained at 37 °C lead to consider *A. equina* mucus an interesting prospect for future biotechnological applications of pharmaceutical and marine technology interest. With regards to pharmaceuticals, the increasing development of bacteria resistant to traditional antibiotics has reached alarming levels, and thus there is the need to develop new antimicrobial agents. In this framework, lysozyme was recently selected as a model protein to develop more potent bactericidal agents thus introducing, a new conceptual employment of lysozyme [69]. Lastly, the antibacterial proteins of *A. equina* mucus could be used to deter the settlement of bacteria representing the primary colonizers in the development of marine biofouling thus constituting an alternative natural antisettlement agents compared to the banned paints and organic biocides [69]. In this framework, it is intriguing that lysozyme-like proteins have also already been evidenced in other cnidarians [121,122].

## 7. Conclusions

Comparative immunobiology studies have led to the abandonment of the idea that invertebrates do not possess immune capacity. Cnidarians possess components of the main routes of immunity of invertebrates. The receptors and pathways already identified indicate that these basal invertebrates are far from being “simple” in the range of methods they have to deal with potential germs and pathogens.

Cnidarian-associated microbial communities are probably a result of a functional cross-talking because cnidarian need to control the resident beneficial microbes, not as a response to invasive pathogens, but because, just as black can exist only if white is visible, so too the use of the same thrifty ways for distinguishing pathogens could be considered the possible origin of the first immunity arms.

In Cnidarians, the crucial activities in structuring tissue-associated microbial communities, useful to the animal’s health, are related to the increasing evidence of the existing innate immune responses involved in the disruption of harmful microorganisms. The present review represents a contribution to reduce the gaps in the current knowledge, regarding the complex relationships established between cnidarians and microorganisms, as well as to provide an overview of the potential biotechnological applications of the defensive compounds present in these invertebrates.

## Figures and Tables

**Table 1:** Coral microbial pathogens.

Disease	Pathogen	Coral Host
Black band	*Roseofilum reptotaenium, Desulfovibrio, Beggiatoa* sp.	Several
White band I	Gram (-) bacterium	Several
White band II *	*Vibrio carchariae*	*Acropora* sp.
Aspergillosis *	*Aspergillus sidowii*	Gorgonians (sea fans)
White pox *	*Serratia marcescens*	*Acropora palmata*
Bleaching *	*Vibrio shiloi*	*Oculina patagonica*
Bleaching and lysis *	*Vibrio corallilyticus*	*Pocillopora damicornis*
Yellow blotch	*Vibrio alginolyticus*	*Monastraea* sp.
Red band	*Oscillatoria* sp. and other cyanobacteria	Several
Dark spots I	*Vibrio* sp. ?.	Several
Dark bands	?	Several
White plague (Eilat)	*Thalassomonas loyana*	Several
White plague	*Aurantimonas coralicida*	Several
White plague I	Gram (-) bacterium	Several
*Porites* ulcerative white spots	*Vibrio* sp.	Several

* Koch′s postulates fulfilled.
